# Using the suicide audit method to examine trajectories of adolescent girls following a suicide attempt in Québec, Canada: a study protocol

**DOI:** 10.1136/bmjopen-2025-115584

**Published:** 2026-04-02

**Authors:** Toula Kourgiantakis, Éric Mercier, Mélissa Côté, Romy-Naïma Tousignant, Audrey-Anne Dumais Michaud, Nathalie Maltais, Jessica Rassy, Alain Lesage

**Affiliations:** 1 École de travail social et de criminologie, Université Laval, Québec City, Québec, Canada; 2 Centre de recherche du CHU de Québec, Université Laval, Québec City, Québec, Canada; 3 Faculté des sciences de l’éducation, Université Laval, Québec City, Québec, Canada; 4 Département des sciences de la santé, Université du Québec à Rimouski, Rimouski, Québec, Canada; 5 École des sciences infirmières, Université de Sherbrooke, Sherbrooke, Québec, Canada; 6 Département de psychiatrie et d’addictologie, Université de Montréal, Montreal, Québec, Canada

**Keywords:** Adolescent girls, Family, Suicide & self-harm, Suicide Audit, Qualitative Research, Public Health

## Abstract

**Abstract:**

**Introduction:**

Suicide is a major public health concern among youth in Canada and worldwide. The most rapid increases in suicidal ideation, self-harm, and suicide attempts have been observed among adolescent girls, particularly since the COVID-19 pandemic. Recent studies report disproportionately high rates of emergency department visits and hospitalisations for suicide-related concerns among adolescent girls. Despite these concerning trends, limited evidence exists on the life trajectories, needs, and service pathways of adolescent girls who attempt suicide. This protocol describes a qualitative suicide audit focused on adolescent girls aged 12–17 who were hospitalised following a suicide attempt in two regions of the province of Québec, Canada. The aim is to understand developmental trajectories, document services received and identify individual, relational and systemic factors influencing these trajectories to generate recommendations that inform suicide prevention.

**Methods and analysis:**

Using a narrative qualitative design and a community-based research approach, data will be collected from semi-structured interviews with adolescents and parents, parent questionnaires and hospital health records. These data will be integrated to develop anonymised case vignettes. A multidisciplinary panel, including clinicians, health system stakeholders, community partners and individuals with lived experience, will review each case to identify gaps and strengths in care and generate case-level and cross-case recommendations for clinical practice, health policy and professional training.

**Ethics and dissemination:**

Ethics approval was obtained from the research ethics committee (REC) of the Centre intégré de santé et de services sociaux de Chaudière-Appalaches, which serves as the reviewing REC, with administrative reviews underway at two other health authorities. Findings will be disseminated through peer-reviewed publications, conference presentations and collaborative knowledge-mobilisation activities with clinical and community partners, including practice-oriented tools and accessible materials for adolescents and parents.

**STRENGTHS AND LIMITATIONS OF THIS STUDY**

- The study uses a suicide audit methodology to examine the trajectories of adolescent girls who have attempted suicide within a Canadian context.
- The design incorporates triangulation across data sources, data collection methods, and multidisciplinary investigators.
- An anonymised case vignette for each adolescent will be systematically reviewed by a multidisciplinary expert panel using an unmet-needs framework to formulate practice, policy, and professional training recommendations.
- The study is conducted in two regions of Québec, Canada, which may limit transferability to other jurisdictions.
- Focusing solely on adolescent girls provides an in-depth understanding of their trajectories and experiences but does not capture the experiences of boys, transgender, or non-binary youth.

## Introduction

Suicide is the second leading cause of death among youth and young adults aged 15–34 in Canada and remains an urgent public health concern.^1^ Among youth in Canada aged 10–19, the suicide rate increased from 5.7 to 6.2 per 100 000 between 2020–2021 (+8.8%), rose slightly to 6.4 in 2022 (+3.2%) and then declined to 5.3 in 2023 (−17.2%).^1^ While this recent decrease in suicide is encouraging, there has been a notable increase in self-harming behaviours, suicidal ideation, and suicide attempts among adolescents. A recent Canadian study reported that 14.1% of Canadian youth aged 15–17 experienced suicidal ideation in the past year and 6.8% reported a lifetime suicide attempt.^2^ Concerning trends have also been observed among adolescent girls in both Canada and the USA.^3 4^ In Canada, the suicide rate for boys aged 10–19 is 1.5 times higher than that of girls, and for young men aged 20–34 three times higher.^1^ However, among adolescent girls aged 10–19, suicide rates have risen consistently from 2011 to 2023, while rates among adolescent boys have declined or stabilised.^5^

Girls aged 10–19 have the highest rates of hospitalisation for intentional self-harm in Canada (239.6 per 100 000), more than four times the rate of boys of the same age and significantly higher than any adult age group.^6^ Among adolescents aged 15–19, the lifetime prevalence of suicide attempts is 7%, which is five times that of boys and exceeds the rates observed among women and men across all age groups. Suicidal ideation is also highest among girls aged 15–19, with 19.6% reporting serious thoughts of suicide, which is more than double the proportion observed among boys (9.1%) and higher than all adult age groups.^6^

A recent public health report on suicide from the Canadian province of Québec indicates that adolescent girls show the highest and most concerning levels of suicide-related service use across all indicators, including emergency department visits for suicidal ideation and suicide attempts, as well as hospitalisations for suicide attempts.^7^ Emergency department visits for suicidal ideation are elevated among girls aged 15–19 (1405.4 per 100 000 in 2024), and although rates have begun to decline slightly since 2021, they remain far higher than those observed among boys and adults. Emergency department visits for suicide attempts also reveal marked increases among girls, particularly those aged 15–19, whose rates have risen steadily since 2016 to reach the highest levels of any age or gender group, while rates among boys have remained low or stable. Hospitalisations for suicide attempts follow a similar pattern: rates among girls aged 10–14 have tripled since 2010, and girls aged 15–19 continue to have the highest hospitalisation rates despite recent declines.^7^ These trends highlight a pronounced and persistent gender disparity, with substantially higher levels of suicide-related service use among adolescent girls compared with boys and other age groups.

Most youth and young adults who attempt suicide do not receive the services and treatment they need.^8^ As a result, emergency departments play a central role in crisis care and frequently serve as a first point of contact for youth experiencing distress.^9^ Chiu *et al*^10^ found that between 2009–2017, mental health-related visits among youth in the Canadian province of Ontario increased by 89%, with the largest increases among adolescents aged 13–17 and young adults aged 18–20. Anxiety and mood disorders were highly prevalent, along with rising rates of high-acuity mental health and addiction-related visits. The authors noted that outpatient mental health service use increased only modestly and that most youth did not receive timely follow-up care before or after an emergency department visit. Individuals with suicidal behaviours often seek help in hospital emergency departments, and research shows that rapid and intensive follow-up is essential once someone leaves hospital or is discharged.^11^ Studies show that suicide risk is highest within 3 months after discharge.^11 12^ Canadian research also indicates that more than half of individuals who died by suicide had visited an emergency department within the previous year, and 10% had done so in the month before their death.^13 14^ However, the level and consistency of care provided in emergency departments do not always match these elevated risks, with considerable variability across settings.^13 15 16^ Rassy *et al*^16^ examined the quality of care provided to adults who had attempted suicide and identified several gaps, most notably in coordination and continuity of care. The authors reported that only 52% of individuals presenting with a suicide attempt had a documented suicide risk assessment in their medical chart. Another study examining the experiences of patients who visited the emergency department for a suicide attempt or suicidal ideation found that many described the experience as distressing and inadequately responsive to their needs.^17^

These trends are particularly concerning given the close relationship between mental health concerns and suicide risk. Studies consistently show that approximately 90% of individuals who die by suicide have a mental health or substance use-related concern.^18^,^20^ A global study examining youth suicide trends from 1990 to 2020 found that suicide risk is ten times higher among young people with mental disorders.^21^ In Québec, a survey of high school students showed that levels of psychological distress increased for all youth between 2010–2017 and were twice as high for girls (40%) as for boys (19%).^22^ In the neighbouring province, the Ontario Student Drug Use and Health Survey reported an increase from 24% of high school students reporting moderate distress in 2013 to 51% reporting moderate to serious levels of psychological distress in 2023. Serious distress rose from 11% in 2013 to 27% in 2023. This survey did not report gender differences.^23^

Suicidal ideation, previous suicide attempts, and self-harm are serious risk factors for suicide.^19 21 24^ The most recent public health report on suicide in Québec indicates that suicide attempts remain a considerable public health concern, particularly among adolescent girls, who are identified as a vulnerable group. The analysis further indicates that emergency department visits for suicidal ideation or suicide attempts constitute critical points of contact within the healthcare system. These visits offer important opportunities to identify youth in distress, ensure appropriate referral and provide timely support.^7^ Given this context, it is essential to better understand the factors contributing not only to suicide but also to suicidal ideation, previous attempts, and self-harm.^25^ Suicide audits offer one way to do so, providing a structured review process that contributes to quality improvement.^26 27^ Suicide audits also offer an important means of identifying risk and protective factors across multiple levels, including individual, family, community, societal, cultural and structural domains.^27^ The value of this approach is reinforced by the recent public inquiry on suicide in Québec, where the coroner formally recommended that all health and social service institutions implement systematic suicide audits to better identify gaps and improve suicide prevention practices.^28^

Suicide audits also highlight the essential role of parents, who act as key informants by providing critical developmental and contextual information and serve as central partners in prevention. Several audits point to gaps in communication with families, limited parental involvement in follow-up planning and unmet needs for guidance and support, particularly during and after emergency department visits.^19 26 29^ A qualitative study with bereaved parents further indicates that families offer crucial insights into youth trajectories and systemic failures, yet frequently experience exclusion from services and decision-making, underscoring the importance of integrating parental perspectives into suicide audits and suicide prevention efforts.^30^ Another recent study of parents of adolescents with suicide attempts found that parents often assume a central case management role and are well positioned to identify adolescents’ needs, as well as gaps in care and service delivery.^31^ Suicide audits also make it possible to examine adolescents’ trajectories across different stages of development, helping to identify needs that were or were not met by various systems^29^ and to generate recommendations through multidisciplinary panel reviews that evaluate gaps in care and propose improvements in services, training, and policies.^26 27^

Although suicide audits offer important benefits, their use in Canada has been inconsistent, and most audits conducted to date have focused on adult populations.^18 26 27^ Renaud *et al*^19^ conducted a suicide audit focused on youth in Québec, examining 67 cases of adolescents and young adults aged 13–25. The data were collected between 2002–2005, and the sample was predominantly male (80%), with 65% classified as young adults. Youth who died by suicide had complex clinical and psychosocial profiles, most often involving substance use and mood disorders, interpersonal distress and prior suicide attempts. Despite these complex needs, service use in the year preceding death was limited, and when services were accessed, needs were frequently insufficiently addressed. The authors recommended improved coordination and continuity of care, mental health promotion and training for service providers, development and implementation of systematic protocols across the province, and improved availability and access to services and treatment. In summary, evidence specific to adolescent girls remains limited, despite recent data indicating that they are experiencing the most concerning increases in suicide attempts.

This qualitative study aims to address these gaps by better understanding the trajectories, needs, and services of adolescent girls aged 12–17 who have attempted suicide and have been hospitalised in two jurisdictions in the province of Québec. It also seeks to generate recommendations to enhance suicide prevention for adolescent girls. The specific objectives of this study protocol are as follows: (1) examine the life trajectories of adolescent girls to better understand their needs, vulnerabilities, and risk and protective factors; (2) analyse the health and social services they received throughout their trajectory; (3) formulate recommendations to improve suicide prevention among adolescent girls aged 12–17.

## Methods and analysis

### Study design

This study uses a narrative qualitative design, which aims to understand participants’ stories, experiences, and trajectories over time.^32^ The study is also informed by a community-based research approach, which is a collaborative, partnership approach to research that seeks to increase knowledge and understanding of a phenomenon in order to inform changes to policy and practice.^33^ Community-based participatory research is particularly relevant in public health, as it attends to multiple determinants of health, including biomedical, psychological, social, economic and cultural dimensions. It involves an interdisciplinary team of researchers and community partners who contribute to all aspects of the research process, including defining the problem, collecting and interpreting data, disseminating findings and applying results to address community issues.^33^ This qualitative study protocol is reported in accordance with the Standards for Reporting Qualitative Research (SRQR).^34^ See online supplemental file 1 for the completed SRQR checklist.

Our community partners include the Centre de Prévention du Suicide de Québec, the first suicide prevention community organisation established in Canada, which offers prevention, intervention and postvention services to those affected by suicide.^35^ Another partner is the Regroupement des Centres de Prévention du Suicide, a provincial organisation that supports local suicide prevention centres across the province. We are also collaborating with the Association québécoise de prévention du suicide, a provincial organisation that coordinates and supports suicide prevention initiatives, including public education campaigns and training for practitioners.^36^ The research team is a diverse interdisciplinary group composed of researchers and clinician-scientists from social work, nursing, psychoeducation, emergency medicine, and psychiatry.

### Setting and context

This study is being conducted in Québec, a French-speaking province of Canada with a population of approximately nine million residents. The province is divided into 17 administrative regions that organise the delivery of government services, including education, healthcare and social services.^37^ This study will take place in two of these regions: Capitale-Nationale (region 03), which includes the large urban area of Québec City, and Chaudière-Appalaches (region 12), located on the south shore of the St Lawrence River and characterised by a mix of rural and small-town communities that are geographically dispersed. Chaudière-Appalaches is among the regions with higher suicide rates in the province (17.5 per 100 000 people), while Capitale-Nationale has a rate of 13.6 per 100 000, slightly above the provincial adjusted rate of 12.5 per 100 000.^7^

### Study sample and recruitment

The sample will include adolescent girls aged 12–17 who were hospitalised following a suicide attempt within the past 3 months in the Québec City or Chaudière-Appalaches regions, as well as their parents or caregivers. In narrative inquiry studies, sample sizes typically range from 5 to 30 participants or continue until data saturation is reached.^38 39^ We will aim to recruit 20 adolescent girls from each region, for a total of 40 adolescents and approximately 40 parents or caregivers. The number of parents or caregivers may be higher if more than one parent from the same family chooses to participate. Purposeful sampling will be used to recruit participants who share relevant characteristics and lived experience of the phenomenon being studied.^40^ Parents and adolescents will be recruited through three hospitals (two hospitals in region 03 and one hospital in region 12) that treat adolescents who have attempted suicide.

### Procedures

This study is being conducted in close collaboration with clinical teams in the participating hospitals. At the discharge planning meeting, a key member of the clinical team, such as the chief paediatrician, chief child psychiatrist, chief of the youth clinical team or nurse manager, will introduce the study and ask parents whether they authorise the clinical team to provide their contact information to the project coordinator. The clinical team will then forward the authorisation form, including the parents’ contact information, to the project coordinator, who will contact the parents to schedule a meeting and present the research study. Parents who agree to participate will sign a consent form for themselves and for their adolescent. Once parental consent is obtained, the researcher will explain the study to the adolescent and obtain her assent.

Parents and adolescents will participate in separate interviews at a location that is most convenient for them, such as their home, the research lab at Université Laval or online via a virtual meeting platform. The interviews will be led by the principal investigator (TK), who is an experienced family therapist and social worker with training in suicide intervention. She will be accompanied by the project coordinator (R-NT), who is a graduate student with suicide intervention training. Parent interviews are expected to last approximately 2–3 hours and may be completed in one or two sessions. Interviews with adolescents are expected to last 1–2 hours and may also be conducted in one or two sessions. All interviews will be audio-recorded for transcription. Both parents and adolescents may stop the interview at any time. Parents will also complete an online questionnaire prior to their interview. The project coordinator will extract key information from the adolescent’s hospital health record.

Adolescents will receive a $C60 gift card for participating in the study. Parents will receive $C60 for completing the online questionnaire and $90 for taking part in the interview. If the interview is conducted in two sessions, the compensation will be divided between them.

### Data collection

Data collection is estimated to take place from January 2026 to December 2026. The study draws on three sources of information: (1) semistructured interviews with adolescents and parent(s); (2) a questionnaire completed by parents; and (3) information obtained from the adolescent’s hospital health record.

The semistructured interview guides for parents and adolescents are adapted from the Life Trajectory Calendar,^41^ which has been used in previous suicide audits.^29 42^ The questions follow a life-course calendar designed to reconstruct an adolescent’s trajectory since birth across multiple domains and developmental stages, including family, interpersonal relationships, housing, significant life events, adversities, mental and physical health, substance use and other addiction-related concerns, mental health and social services, school, social and protective factors, the suicide attempt, the hospitalisation experience, and continuity of care postdischarge.^42^

The online questionnaire completed by parents prior to their interview is adapted from the National Confidential Inquiry into Suicide and Safety in Mental Health, a validated instrument that has collected in-depth information on all suicides in the UK since 1996.^43^ This ongoing clinical audit has informed the development of a toolkit containing recommendations related to key elements of quality and safety of care. Evidence indicates that regions implementing these recommendations more consistently have observed a reduction in suicide rates.^44^ The parent questionnaire includes seven sections addressing family situation and housing; the adolescent’s mental and physical health during childhood and adolescence; school environment; employment and activities; adversities or difficult experiences; services and treatments received prior to the most recent hospitalisation; the most recent hospitalisation related to the suicide attempt; and services received following that hospitalisation. The questionnaire adapted for this study will be administered online via LimeSurvey prior to the interview.

Information from the adolescent’s hospital health record will be collected using an adapted quality-of-care checklist developed by France’s Agence nationale d’accréditation et d’évaluation en santé.^45^ This checklist has been used in a previous suicide audit with adolescents,^46^ and in research examining quality of care following a suicide attempt.^16^ The adapted checklist consists of 21 items assessing emergency department care, psychiatric follow-up, coordination of care, documentation of suicide risk assessment, family history, continuity of care after discharge, assessment and treatment planning for substance use if needed, presence of a safety plan, transfer of notes to the family physician, diagnoses, medications and hospitalisation history. 

### Data analysis plan

Data analysis will follow a two-stage process integrating three sources of data: (1) semistructured interviews with adolescents and parents; (2) parent questionnaires; and (3) hospital health records. First, all interviews will be transcribed verbatim and anonymised. Data from interview transcripts, parent questionnaires and hospital records will then be systematically reviewed, extracted and triangulated. Relevant information from each source will be analysed and coded to construct a comprehensive, anonymised case vignette that reconstructs each adolescent’s developmental trajectory, including psychosocial and family context, mental and physical health, substance use, school and social functioning, service utilisation, the suicide attempt, hospitalisation and postdischarge follow-up.

The second stage involves a multidisciplinary panel review of the anonymised case vignettes. The panel will include clinicians and researchers with expertise in social work, nursing, psychiatry, emergency medicine, and psychoeducation, as well as people with lived experience and policy or decision-makers involved in suicide prevention and youth mental health. The panel will analyse each case by comparing the care received with evidence-based standards of practice to determine whether identified clinical and service needs were met or unmet, following the methodology described by Lesage *et al*^27^ and based on the unmet-needs framework developed by Brewin *et al*^47^ Within this framework, a need is considered present when a clinically significant problem exists for which an effective intervention is available, and unmet when such an intervention was not provided or was inadequate. The panel will identify clinical, organisational, and systemic gaps in care and formulate case-specific preventive recommendations. A cross-case qualitative synthesis will then be conducted to identify recurring patterns of service gaps and strengths in care and service organisation. Through an iterative consensus process, recommendations will be consolidated into three levels of action: clinical practice, policy and organisation of services, and education and professional training. There will be a total of eight panels, and each will review five vignettes.

Several strategies will be used to enhance rigour and trustworthiness. These will include triangulation of multiple data sources (interviews, parent questionnaires and hospital records), researcher triangulation through multidisciplinary panel review, prolonged engagement with the data during case reconstruction and cross-case synthesis, systematic documentation of analytical decisions and maintenance of a clear audit trail to ensure transparency and consistency across cases.^48^ Reflexivity is an important component of data analysis and recognises researchers’ subjectivity as integral to the analytical process. Reflexivity involves ongoing self-examination of how researchers’ positions, assumptions and professional experiences may shape interpretation. Attention will be given to how disciplinary perspectives within the multidisciplinary panel may influence judgments about service adequacy and standards of care, and divergent interpretations will be explored prior to reaching consensus.^49^

### Patients and public involvement

We formed an advisory committee that includes parents and young adult women with lived experience, as well as a clinician recruited through a community suicide prevention centre. The committee was established shortly after the study was funded and before submission to the ethics committee. Members contributed to reviewing study procedures, measures and materials, and provided feedback on the feasibility and acceptability of the research process. The advisory committee will remain involved throughout the study through three to four meetings per year and additional communication by email. They will also be consulted on plans for sharing study results with participants and the broader community. Advisory committee members will receive an honourarium, provided as gift cards, in recognition of their time and contributions.

## Ethics and dissemination

The study will be conducted in three hospitals, each affiliated with a distinct administrative health authority. Two hospitals are located in the Capitale-Nationale region and one in the Chaudière-Appalaches region. In Québec, a research project conducted simultaneously in multiple hospitals is classified as a multicentre study. Multicentre studies require a centralised ethics approval process overseen by a research ethics committee (REC) and complemented by local administrative review at each participating site.^50^ The REC of the Centre intégré de santé et de services sociaux de Chaudière-Appalaches (CISSS-CA) serves as the reviewing REC, and ethics approval has been obtained from the CISSS de Chaudière-Appalaches (protocol number MP-23-2026-1274). Administrative reviews are underway for the two hospitals in the Capitale-Nationale region, one affiliated with the Centre intégré universitaire de santé et de services sociaux de la Capitale-Nationale and the other with the Centre hospitalier universitaire de Québec-Université Laval.

This study includes a suicide audit focused specifically on adolescent girls, which will provide important insights into their trajectories and service needs and contribute to understanding patterns in youth suicide observed both nationally and internationally. Study findings will be shared with scientific, clinical, community and policy audiences through peer-reviewed publications, conference presentations and collaborative knowledge-mobilisation activities with our partner organisations. We will also work with provincial partners and members of our advisory committee to develop practice-oriented tools specific to adolescent girls, as well as accessible materials for adolescents and their families. Results will be disseminated to participating institutions and the broader public through webinars, professional networks and community organisations involved in suicide prevention.

## Supplementary material

10.1136/bmjopen-2025-115584 online supplemental file 1
